# Case Report: Kidney injury following non-medical massage

**DOI:** 10.3389/fneph.2026.1730580

**Published:** 2026-02-06

**Authors:** Michael Hieu Truong, Vanessa Quynh Tran, Spencer Doan Tran, Calvin Tuan Nguyen, Phuong Chi Pham, Quynh Giao Pham

**Affiliations:** 1 Chicago Medical School at Rosalind Franklin University of Medicine and Science, North Chicago, IL, United States; 2 University of California, Los Angeles, Los Angeles, CA, United States; 3 University of Southern California, Los Angeles, CA, United States; 4 Virginia Commonwealth University School of Medicine, Richmond, VA, United States; 5 David Geffen School of Medicine at University of California, Los Angeles, Los Angeles, CA, United States; 6 VA Greater Los Angeles Healthcare System, Los Angeles, CA, United States

**Keywords:** case report, deep tissue massage, kidney infarction, low back pain, massage therapy

## Abstract

**Background:**

Deep tissue massage is widely used for musculoskeletal pain and is generally considered safe. We report a case of acute kidney injury following non-medical deep tissue massage.

**Case presentation:**

A 50-year-old healthy man presented with acute right lower quadrant abdominal pain 4 h after receiving a deep tissue massage for low back pain from an unlicensed therapist. CT angiography revealed a segmental acute infarction of the right kidney with occlusion of the superior segmental artery. The patient was successfully treated with anticoagulation therapy with gradual resolution of symptoms.

**Conclusions:**

This case highlights the potential for severe vascular complications from deep tissue massage, even in healthy individuals. The proposed mechanism is direct mechanical trauma to the renal artery. Prompt evaluation of abdominal pain following massage is essential.

## Introduction

Low back pain is a common pain disorder that affects well over 40% of US adults aged 45 years or older (1). Prior to obtaining formal medical evaluations, many patients seek non-medical interventions for symptomatic treatment (2). Generally, massage therapy is considered safe, without significant side effects, while benefits include improved joint range of motion, increased muscle compliance, and overall relaxation responses (3).

Until recently, the use of massage to treat musculoskeletal pain has been considered a traditional practice worldwide and often performed by non-licensed individuals. Currently, many countries have begun establishing licensing requirements for massage therapy. In the United States, individual states are charged with regulating massage therapists, and five states still do not require any form of formal massage therapy training or certifications (4). Although some studies question the effectiveness of massage therapy, this modality remains a widely accepted and readily accessible modality for quick symptomatic relief of pain (5).

## Narrative

The patient was a healthy 50-year-old Asian man with no prior existing medical conditions who sought massage therapy for general relaxation and mild low back spasm pain. This pain occurred after a sports injury 1 month prior; the patient had fallen off his skateboard onto his buttocks, incurring moderate pain in his lower back area immediately after the fall. At the time of the initial injury, the pain was described as a localized aching sensation of 6/10 intensity over bilateral lumbar paraspinals, which worsened with the bending and twisting of the back. There was no pain radiation or associated symptoms such as fever, chills, malaise, or weakness. The patient did not seek any medical evaluation for the pain. After taking an over-the-counter non-steroidal anti-inflammatory drug (NSAID) for several days, the back pain gradually subsided over 2 weeks without any additional treatment. However, the patient noted that the back pain returned occasionally following strenuous weightlifting exercises.

While abroad on vacation 1 month after the initial skateboarding incident, the patient opted to undergo a non-medical massage session focusing on the lumbar paraspinal muscles, completed by an unlicensed massage therapist. Four hours after the massage session, he noticed intense right lower quadrant abdominal pain. The pain was described as a localized aching deep pain of 6/10 intensity without radiation. Within the next 2 h, this pain worsened to 8/10 in intensity, prompting a visit to the emergency room for evaluation. The patient denied fever, chills, diaphoresis, nausea, vomiting, diarrhea, flank pain, dysuria, hematuria, shortness of breath, or palpitation. He was not taking any medications during this time and exercised regularly.

The patient underwent surgical repair of a traumatic right lateral pectoralis tear a year prior, but had no other surgical history. There was no significant past medical history (hypertension, diabetes mellitus, hypercholesterolemia, or kidney, gastric, pulmonary, or cardiac conditions) nor psychiatric history reported. Socially, he was married with three daughters, ages 8, 5, and 2. He completed a medical doctorate degree and worked in a leadership position at a privately owned company. He drank only socially, never smoked, and never used illicit drugs. His father had type II diabetes, and his mother had hypertension. There was no family history of heart disease, cancer, stroke, or hypercoagulable conditions.

**Figure 1:**
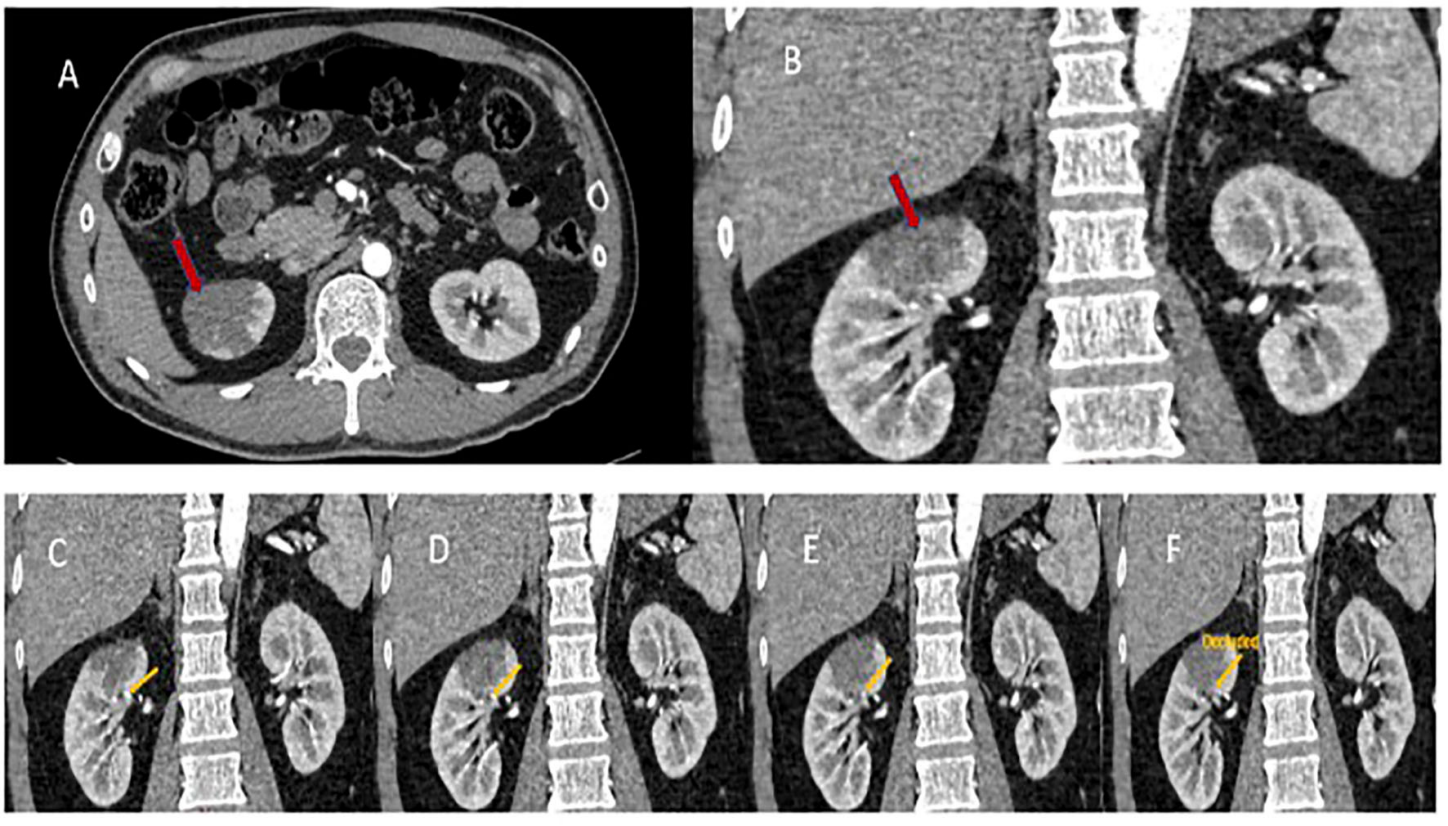
CT angiogram of the pelvis. CT angiogram of the pelvis showing areas of decreased attenuation (red arrows) on the axial **(A)** and sagittal **(B)** views, signifying infarction of the right kidney upper pole. Sequential flow of contrast **(C–F)** shows the absence of contrast blood in the proximal renal artery branch supplying the upper pole of the right kidney (yellow arrow), indicating occlusion of the artery.

**Figure 2:**
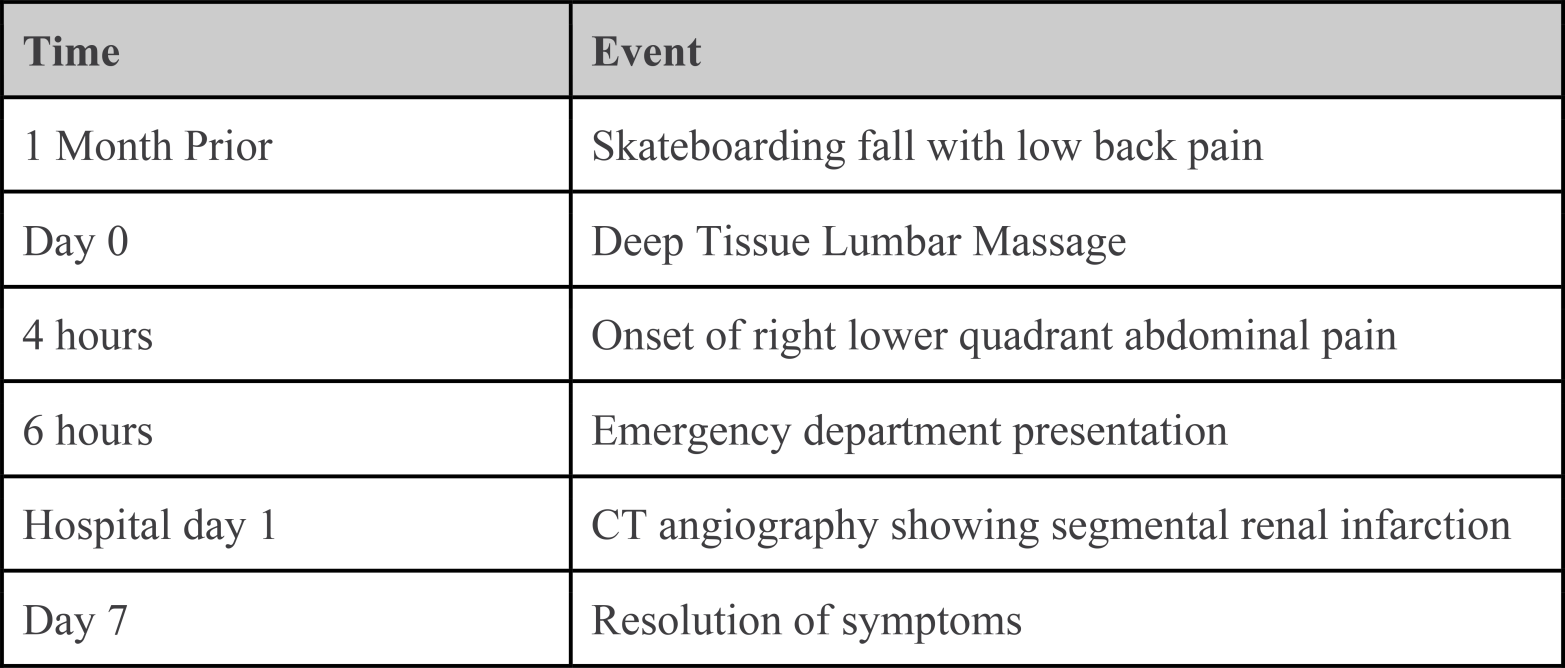
Timeline of clinical events. Key events from initial low back injury through massage exposure, symptom onset, diagnostic imaging, and recovery.

His physical exam revealed a non-distended abdomen with localized right lower quadrant abdominal pain with palpation. No lower extremity edema, calf tenderness, or asymmetry suggestive of deep vein thrombosis was noted. Vital signs and routine lab work, which included CBC, chemistry, liver panel, amylase, creatinine kinase, and coagulation panel, were all within the normal range. Electrocardiogram, echocardiogram, and Holter monitoring screenings were performed and indicated no abnormalities. An initial abdominal CT scan did not reveal any abnormalities, but a triple-phase contrast-enhanced CT angiogram of the abdomen revealed demarcated hypoattenuation of the upper pole of the right kidney parenchyma, consistent with segmental acute infarction (**Figure 1**). Further evaluation of the CT angiogram revealed a flow disruption of the right superior segmental artery, consistent with a thrombotic or embolic event. CT angiography did not demonstrate features suggestive of fibromuscular dysplasia, including arterial beading or focal stenoses, making spontaneous arterial dissection less likely. The right kidney was supplied by a single renal artery with a discrete occlusion of the superior segmental branch, which anatomically corresponded to the infarcted upper pole. The patient was treated with anticoagulation therapy with low molecular weight heparin and pain medications. One day later, he developed a low-grade fever, chills, and general malaise attributed to post-embolic/thrombotic syndrome. His constitutional and abdominal pain symptoms gradually resolved over 7 days (**Figure 2**).

## Discussion

Massage therapy is generally safe, with few significant adverse effects ever having been reported in contemporary literature (6). The benefits of massage therapy include increased local tissue blood perfusion to increase muscle relaxation, improved flexibility, and reduced muscle tension. Although there is no commonly accepted definition of “deep tissue massage,” some authors define therapeutic massage as involving effleurage, petrissage, tapping, and friction, while defining deep tissue massage as involving oblique pressure (using lengthening and cross-fiber strokes) that may affect deeper muscles and tissues (7, 8). A literature review by E. Ernest reported injuries from different types of massages to various locations in the body (9). These included massages given by both professionals and laymen, with and without the use of professional equipment. Many of the reported complications with massages were seen in patients with preexisting conditions, such as thromboembolism in a patient with deep vein thrombosis, hematoma in a patient on anticoagulation therapy, a dislodged thrombus in a patient who received aortobifemoral bypass surgery, and kidney rupture in a patient with hydronephrosis (6, 10). With respect to vigorous deep back massages, previously reported complications included thigh hematoma, buttock hematoma, venous thrombus embolism, and acute spinal subdural hematoma (9, 11–13).

To date, there are no reported cases of renal infarction following massage therapy in a patient with no preexisting conditions. Mikhail et al. reported renal artery embolization following back massage in a patient with known aortic occlusive disease (22). In contrast, our patient had no preexisting vascular pathology, making this the first reported case of renal infarction following massage in a previously healthy individual. Renal infarction is rare, and this type of injury due to massage therapy is most likely caused by blunt trauma (14). Prior reported cases of spontaneous segmental infarction have been attributed to cardiogenic causes (atrial fibrillation, myocardial infarction, rheumatic mitral stenosis, etc.), systemic conditions (lupus erythematosus, polycythemia vera, etc.), or iatrogenic causes (15). The patient’s cardiac and coagulation screens were negative, thus excluding prior underlying cardiogenic causes and coagulopathy. In this case, the proposed mechanism of injury is local trauma to the vessel wall, which may have resulted in a thromboembolic event. In addition, arterial compliance decreases with age, making the arteries increasingly vulnerable to external force, pressure, and compression (16). It should be noted that while cardiac monitoring was unrevealing, paroxysmal atrial fibrillation cannot be entirely excluded. However, the close temporal association with mechanical trauma and the lack of recurrent embolic events point to a localized vascular injury.

The location of the kidneys in proximity to the lumbar paraspinal muscles allows for the possibility of injury during deep massages. The kidneys are located lateral to the vertebrae at T12 to L3, with the right kidney positioned slightly lower than the left due to the presence of the liver (17). Posteriorly, the kidneys lie beneath the quadratus lumborum muscle, which is often targeted during deep tissue massages and myofascial releases. The nature of the patient’s injury suggests that a deep mechanical pressure during the massage may have directly or indirectly injured the segmental renal artery that supplies the upper pole, causing infarction of the right kidney (**Figures 1A, B**) (18). A serial CT angiogram confirmed the occlusion of the superior segmental artery (**Figure 1C**).

While it is possible that the patient’s prior skateboarding incident may have contributed to the kidney injury, this scenario is unlikely. The pain symptoms incurred from the fall were localized to the bilateral paraspinal areas, without abdominal or flank pain. The initial pain improved with rest and worsened with physical activity, suggesting a musculoskeletal etiology rather than organ injury (19, 20). Furthermore, the back injury was not associated with constitutional symptoms such as nausea, fever, chills, hematuria, general fatigue, and weakness from the presence of rhabdomyolysis, which could be associated with kidney injury (21). The patient only noticed these constitutional symptoms after he presented with right lower quadrant abdominal pain after the massage. Most notably, there was a lack of temporal association between the fall injury and the onset of acute abdominal pain. However, it may be possible that the fall injury resulted in subclinical vessel damage that was worsened by subsequent massage maneuvers.

Several reported complications resulting from vessel damage are listed in **Table 1** (9–11, 13, 22–24). The presentation of these complications varies, and it is important to note that comorbidities may impact the course of complications; however, all reported cases reviewed were associated with atypical pain following massage therapy. Complications due to deep tissue massages are likely underreported (6, 9). First, there is no standardized surveillance system for massage-related adverse events, particularly when therapies are performed outside regulated medical settings. Second, symptoms may be incorrectly attributed to other conditions, such as musculoskeletal strain or gastrointestinal illness. Third, spontaneous resolution of adverse effects may reduce the likelihood that patients seek medical evaluation. Finally, massage therapy is widely perceived as low risk, lowering both patient and clinician suspicion for serious complications. Given the widespread use and accessibility of massage therapy, particularly for low back pain, failure to recognize massage-related injuries may delay diagnosis and management when complications occur. Improved reporting may be facilitated through increased clinician awareness and standardized patient education regarding warning symptoms following massage therapy, such as severe or persistent abdominal or flank pain, hematuria, or fever.

**Table 1:** Reported incidence of injury following massage therapy.

Author (year)	Type of injury/complication	Patient information and comorbidity	Proposed cause	Treatment	Outcome
Dahril, J. et al. (10)	Rupture of the right kidney secondary to urethral obstruction	A 52-year-old man; hydronephrosis with history of prior surgery due to multiple nephrolithiasis	Trauma caused by abdominal and back massage	Emergency laparotomy surgery revealed a ruptured right kidney; a right nephrectomy was performed	The patient recovered in the ICU and was discharged on the 5th postoperative day.
Sharma, I. et al. (9)	Significant hematoma in the medial right thigh. Limited range of motion due to pain	A 69-year-old woman; pneumonia prior to massage and renal insufficiency with routine epoetin injections	Massage over the right medial thigh caused medial thigh pain, swelling, and ecchymosis immediately following the massage	External compression wrap of the right thigh, PRBC transfusion	The patient’s range of motion returned over the course of a week.
Sun, F. et al. (11)	Left buttock hematoma, damaged arteriole in the superficial branch of the superior gluteal artery	A 59-year-old man; calcified blood vessels	Deep tissue massage, causing pain and numbness in the left lower extremity	Endovascular embolization of the artery utilizing a small coil via the femoral artery	Pain was relieved gradually over an unspecified period.
Maste, P. et al. (13)	Acute spinal subdural hematoma with radiating pain down to the bilateral lower extremities and inability to walk	A 41-year-old man; w/out comorbidities	Vigorous Thai massage involving stepping on the back and waist by a therapist, bending, twisting	The patient was placed on bed rest and treated with IV dexamethasone 25 mg with dose tapering of 5 mg per day.	Full motor recovery with only minor sensory deficits after 2 weeks. There were no neurological symptoms 1 year following the incident.
Chen, H. et al. (24)	Small bowel intramural hemorrhage.	A 68-year-old man; 3-year history of atrial fibrillation and cerebrovascular disease treated with warfarin	Abdominal massage, after which the patient experienced persistent abdominal pain, vomiting, and no bowel movements.	The patient was admitted to the hospital; warfarin was discontinued; bowel rest, nasogastric decompression, hydration, vitamin K, fresh frozen plasma, packed red blood cell transfusion, and total parenteral nutrition were administered/performed.	The patient recovered 3 days following the initiation of treatment.
Mikhail, A. et al. (22)	Left renal infarction by acute embolic occlusion	A 59-year-old man; aortobifemoral bypass	Back massage involving walking on the back while the patient was lying in a prone position	The patient was placed on anticoagulation therapy for 6 weeks, after which an operation was performed for exploration of the abdominal aorta, allowing for the replacement of the aortobifemoral graft.	Repeat abdominal CT scan performed 4 months following presentation showed no left kidney defects.
Yeo, T. C. et al. (23)	Large hematoma over the lower back, part of the massaged area	A 62-year-old man; severe aortic regurgitation 5 years prior	Manual massage trauma in conjunction with anticoagulation and salicylate-induced platelet dysfunction	Warfarin dosage was reduced, and the patient received a blood transfusion.	Hematoma resolved gradually over the course of 1 to 2 weeks.

## Patient’s perspective

“It was quite simple; I fell on my butt a month prior, and it tweaked my back. I had some muscle spasms while on vacation and went to get a massage to feel better. I knew something was seriously wrong when the pain was bad after the massage. I thought that I had ruptured an appendix from the massage. But the doctors said that a blood vessel to my right kidney was damaged, so I was given blood thinner to help dissolve the clot and prevent new ones. I feared the long-term consequences from my damaged kidney and did everything that I was instructed to do. There were no side effects from the blood thinners, but the thought of being on it for several months was frightening. Besides having fever, chills, and body ache for several days after I visited the Emergency Department, I felt better after about 1 week.

I have a different perspective about getting massages, which I often get while on vacation. I want other people to be aware about the possible complications from massages. I still think that massages are great for relaxation, but I am now very careful about requesting very intense and deep pressure massages.”

## Limitations

This report establishes an inferential causal relationship between massage therapy and injury based on temporality and exclusion of other causes. No diagnostic angiography was performed initially to characterize the exact nature of the vascular occlusion. In addition, there is a lack of long-term follow-up data on the patient’s renal function, and the contribution from prior blunt trauma cannot be fully excluded.

## Conclusions

Massage therapy is an easily accessible modality for alleviating low back pain. However, caution should be exercised when performing or ordering deep tissue massage therapy; excessive or strong mechanical force has the potential to damage arteries and cause tissue damage, even in healthy individuals with no pre-existing medical conditions. Patients should seek urgent medical evaluation if they develop severe or persistent abdominal or flank pain, hematuria, or fever following massage therapy. In such cases, clinicians should consider prompt evaluation with contrast-enhanced computed tomography, urinalysis, and assessment of renal function to evaluate for possible organ injury. Those who seek massage therapy can limit their risks by seeking out licensed and/or trained massage professionals, while therapists should employ risk-aware techniques, avoid excessive focal pressure over vulnerable anatomic regions, and perform pre-massage screening for recent trauma or underlying medical conditions. It is important to properly communicate to the massage therapist any preexisting injuries as well as any pain experienced during the massage.

## Data Availability

The data analyzed in this study are subject to the following licenses/restrictions: private patient information. Requests to access these datasets should be directed to angelaquynh.pham@va.gov.
